# Roadmap to Catalytic Abatement of Gas Phase Per‐ and Polyfluoroalkyl Substances (PFAS)

**DOI:** 10.1002/anie.202424718

**Published:** 2025-04-07

**Authors:** Patrick Lott, Florian Maurer, Arik Beck

**Affiliations:** ^1^ Institute for Chemical Technology and Polymer Chemistry Karlsruhe Institute of Technology KIT Engesserstr. 18/20 76131 Karlsruhe Germany

**Keywords:** C−F bond, emission control, fluorine, heterogeneous catalysis, PFAS

## Abstract

While the outstanding stability of per‐ and polyfluoroalkyl substances (PFAS) paved the way for their widespread application in a huge variety of applications, it also resulted in their nickname “forever chemicals”. The rising awareness for PFAS‐related environmental and health concerns drives a discussion on the most effective ways to abate PFAS emissions into the environment, i.e. water, soil, and air, and remediation of contaminated matter. In order to address the knowledge gap regarding air pollution by PFAS, this minireview summarizes the current corpus of work in the field and outlines how catalysis can contribute to PFAS abatement in the gas phase. Beyond a mere collection of state‐of‐the‐art knowledge, overarching challenges in catalytic PFAS removal are identified, spanning from fundamental organic and inorganic chemistry, i.e. C−F‐bond activation, to heterogeneous catalysis, i.e. surface reactions at the gas‐solid interface, to reaction engineering, i.e. scaling relations and technical hurdles. In addition, the article introduces concepts and workflows that aim at providing guidance during the design of technological solutions for the efficient control of gaseous PFAS emissions.

## Graphical abstract

In the context of PFAS emission abatement, this minireview addresses catalytic gas phase PFAS decomposition and the associated overarching challenges involving fundamental organic and inorganic chemistry, heterogeneous catalysis, and reaction engineering. In addition, concepts and workflows that aim at providing guidance during the design of technological solutions for the efficient control of gaseous PFAS emissions are introduced.

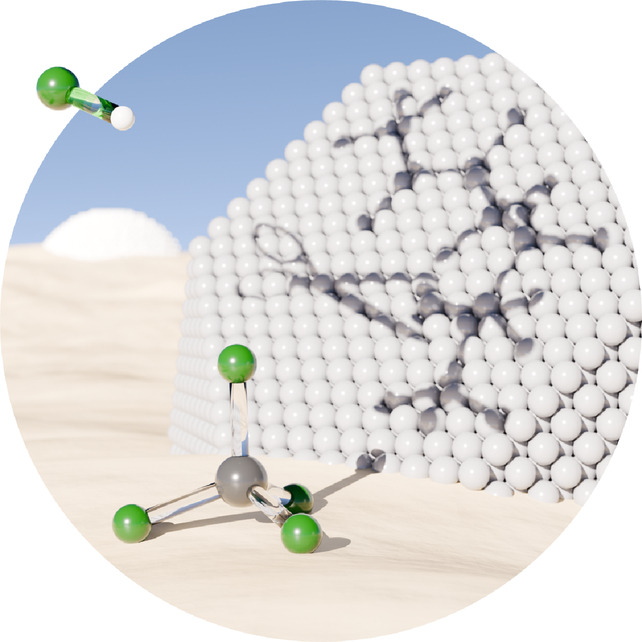

## 1 Introduction

The design of per‐ and polyfluoroalkyl substances (PFAS) enabled the manufacturing of a wide variety of chemically and thermally extremely stable materials:^[1]^ Water‐, oil‐, and dirt‐repellent PFAS are exploited for impregnation of paper and food packaging,[^2^, ^3^, ^4^] the coating of pans and pots for cooking, e.g. with PTFE (polytetrafluoroethylene, commonly known as Teflon®)),^[5]^ and the impregnation of textiles and leather.[^6^, ^7^, ^8^] Also fire extinguishing foams, pharmaceuticals, and pesticides[^9^, ^10^, ^11^] as well as a vast variety of electronic products, in particular lithium‐ion batteries, fuel cells, and photovoltaic solar panels, which are considered crucial for a rapid decarbonization of the global energy system, contain relevant levels of PFAS.[^12^, ^13^, ^14^, ^15^, ^16^] The stability of PFAS arises mainly from the strength of the carbon‐fluorine bond (440 kJ mol^−1^).^[17]^ Their extraordinary stability – natural degradation of PFAS occurs hardly – and extensive use led to accumulation of PFAS in the environment. In the past years, research revealed the extent of PFAS presence in the biosphere,^[18]^ including the human body, and the potential health risks associated to this contamination.[^19^, ^20^] This sparked efforts to remove the current PFAS contamination from the environment, primarily by means of adsorption or direct destruction, and prevent further PFAS emissions.[^21^, ^22^, ^23^, ^24^]

Historically, catalysis has played a pivotal role in emission control and the targeted decomposition of harmful chemicals.^[25]^ In comparison to stoichiometric chemical decomposition,^[26]^ the use of catalysts substantially reduces the needed resources and allows for continuous, long‐lasting emission abatement. Since awareness of risks associated with PFAS has only emerged in recent years, little attention has been paid to the design and fundamental understanding of catalytic PFAS decomposition into benign and/or potentially useful chemicals.

Herein, the type of catalysis and catalytic process relevant to the decomposition of PFAS strongly depends on the source of PFAS emission or contamination, the separation technology, and the PFAS type. While some comprehensive reviews exist on catalytic PFAS decomposition in the liquid phase,[^27^, ^28^] gas phase catalysis has not been addressed in a systematic manner yet. This is especially critical as many of the airborne small chain PFAS have huge global warming potentials of 10’000 (${\mathrm{CF}}_{4}$ ) or 14’000 ($C_{2}F_{6}$ ) ${\mathrm{CO}}_{2}$ equivalents.^[29]^ Uncontrolled emission of gaseous PFAS, therefore, represents another critical accelerator for global warming.

In this paper, we highlight the relevance of gas phase catalysis for PFAS remediation and identify the state‐of‐the‐art. Subsequently, we classify research strategies and the knowledge gaps therein. Beyond providing the reader with a mere literature survey on relevant technologies and methodologies, this articles also aims at shaping and guiding future research.

## 2 Origin and Fate of Gaseous PFAS Emissions

The number of substances classified as PFAS underscores the importance of this substance class: According to the OECD (Organisation for Economic Co‐operation and Development), the group of chemicals considered as PFAS encompasses more than 4’700 different substances,^[30]^ the US EPA (United States Environmental Protection Agency) currently classifies even more than 14’000 chemicals as PFAS‐relevant.^[31]^ Note that in this article, also substances with one carbon atom, such as tetrafluoromethane (${\mathrm{CF}}_{4}$ ), are denoted as PFAS.

**Figure 1:**
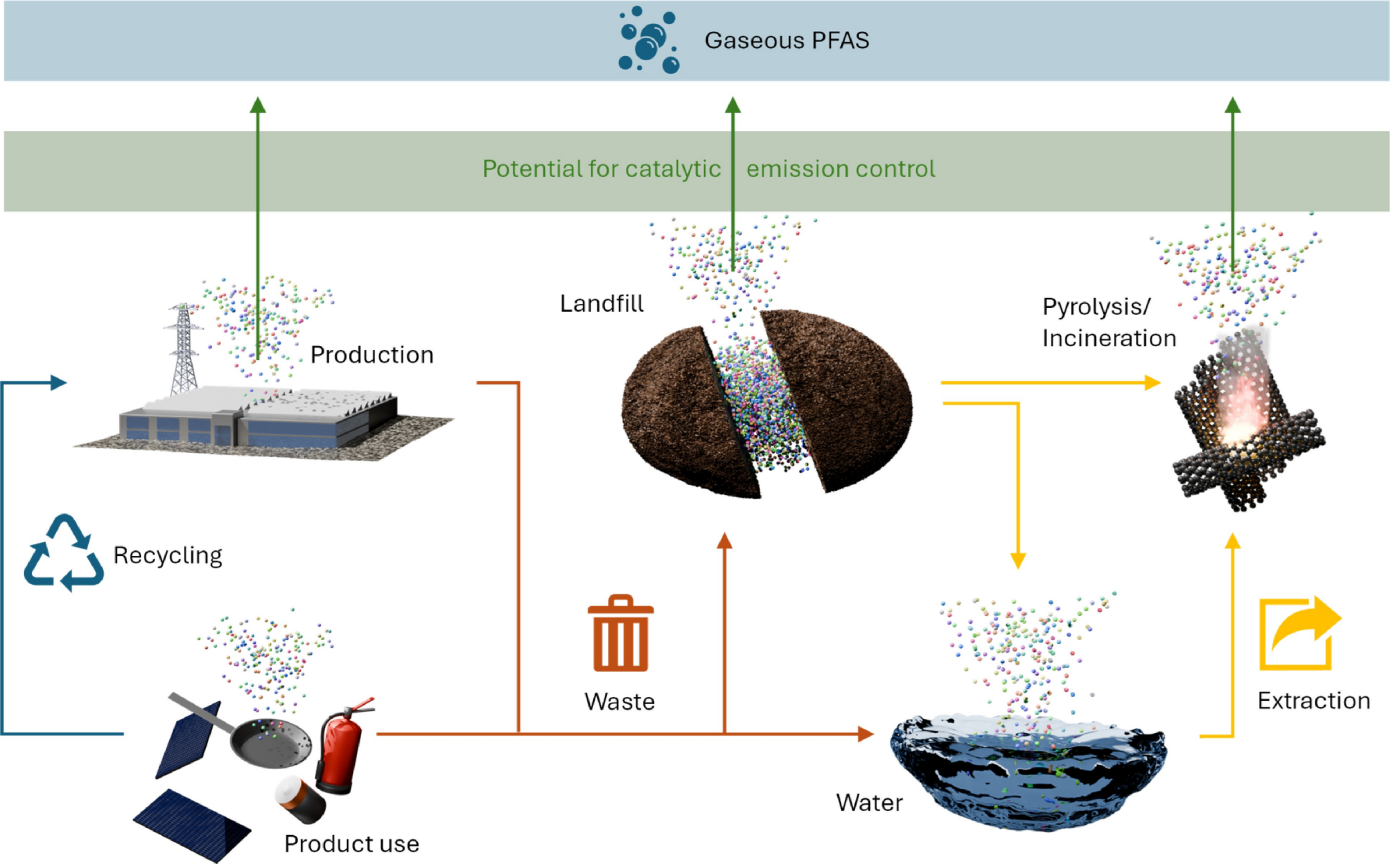
Pathways of PFAS emissions into the gas phase and potential locations of catalytic PFAS emission control application.

Replacing PFAS by alternative chemicals is often challenging or even impossible.^[32]^ Hence, despite increasingly stricter regulation of PFAS production and distribution, a complete ban is expected to result in a major technological gap that cannot be closed yet with state‐of‐the‐art alternatives. Consequently, the inevitability of PFAS makes the control of their emission a timely matter, which due to the large quantities of already emitted PFAS into the environment and the expected significant emissions during the recycling of PFAS‐containing products and waste is relevant even in the unlikely case of a complete ban. In this regard, it is important to mention that emissions of PFAS can occur during their entire lifetime spanning the production of the chemicals and PFAS‐containing products itself, their processing, their recycling, and ultimately their disposal (Figure 1). However, the critical emissions may vary, depending on the production route, the application for which the PFAS is used, the disposal or recycling strategy, and the respective substance itself and its properties, i.e. if the PFAS is solid, liquid, or gaseous. Of course, establishing circularity by re‐use and recycling should always be the primary goal.^[18]^

Outgassing and abrasion of PFAS‐containing products is known to contribute to indoor and outdoor pollution due to gaseous species and particulate matter.^[33]^ However, the ubiquity of products acting as emitters and the comparably low gas phase PFAS concentrations make dedicated emission control measures almost impossible and, above all, uneconomical. Only the expectation of higher PFAS concentrations in (exhaust) gas streams will justify the application of potentially expensive after‐treatment solutions. In this regard, production sites that process fluorinated chemicals are at risk to cause PFAS emissions, among which especially the fluoropolymer industry with its high production volume is an example of great relevance.[^34^, ^35^] Controlling emissions from such industrial processes is expected to be technologically feasible, because production conditions, raw materials, intermediates, and products are typically constant and, thus, the number and type of emitted PFAS is limited.

In contrast, the after‐treatment of off‐gases from landfills, incineration plants, pyrolysis plants, and waste‐to‐energy plants, which can be considered as major PFAS emitters according to a variety of recent studies,[^36^, ^37^, ^38^, ^39^, ^40^, ^41^] will be much more challenging: Since the materials processed in these facilities will strongly vary, the nature of emitted PFAS will be much more diverse. Notably, in the context of fluoropolymer combustion, some studies also concluded that a careful choice of incineration conditions can prevent the emission of undesired PFAS,[^42^, ^43^] which suggests to exploit reaction engineering in addition to catalytic approaches.

The above‐mentioned aspects underscore that the conditions to which PFAS‐contaminated materials are exposed govern both the type of PFAS as well as the pollutant level that ends up in the gas phase. In conclusion, a holistic view on the respective process is necessary, involving the PFAS‐source and the process conditions that are a prerequisite for identifying critical compounds and monitoring their efficient treatment.

## 3 Destructive Technologies Leading to Gaseous PFAS Emissions

In the context of waste management and recycling, two approaches are relevant. Oxidative conditions may be chosen, for instance, in incineration plants and for landfill gas production that commonly relies on biochemical degradation processes. On the other hand, an (almost) oxygen‐free environment can be chosen for pyrolytic processes, such as the (catalytic) pyrolysis of plastic waste[^44^, ^45^] and municipal solid waste.^[46]^ All these processes have to be considered as a potential source of gaseous PFAS emissions that require further treatment, since their feed materials are frequently contaminated with PFAS (Figure 1).

In fact, pyrolysis has been proposed as a feasible treatment option of organic waste, namely, sewage sludge, food, garden, and timber waste, ensuring that a fraction of the original PFAS of less than 3 % is being emitted in the flue gas.^[47]^ Furthermore, Taylor et al.^[48]^ and Gehrmann et al.^[43]^ recently mimicked conditions typical for waste incinerators and concluded that PFAS emissions are very low.

While these findings are encouraging, much broader research is necessary in order to account for the sheer abundance of different PFAS that are used in modern products and their respective chemistry: frequently, decomposition mechanisms are complex and yield a variety of secondary fluorinated products. For instance, by means of infrared spectroscopy, nine prevalent decomposition products evolving in the form of gaseous perfluorocarbon species were identified during the entirely thermal decomposition of solid potassium perfluorooctanesulfonate in the low‐temperature regime from 200 to 550 °C.^[49]^

Under pyrolytic conditions, defluorination, which means the release of inorganic fluorine, was poor. In particular, product formation was found to take place *via* HF elimination, which was observed already at temperatures as low as 200 °C, and relevant levels of thermally stable ${\mathrm{CF}}_{4}$ and $C_{2}F_{6}$ were observed.^[50]^ These results are in line with earlier findings by Weber et al.,^[51]^ who decomposed perfluorooctanesulfonate in an α ‐alumina reactor under pyrolytic conditions; they reported on ${\mathrm{CF}}_{2}$ radicals formation and, among other species, $C_{2}F_{4}$ , HF, and ${\mathrm{SO}}_{2}$ as products. Furthermore, secondary PFAS formation in the form of perfluorocarboxylic acids[^11^, ^52^] has been reported during the incineration of aqueous film‐forming foams, which commonly have been used for firefighting purposes and whose disposal by means of combustion becomes increasingly relevant.

These findings underscore that merely relying on rather simple descriptors, such as the destruction efficiency, is insufficient for evaluating the decomposition of PFAS and can be misleading because also secondary products and, thus, the selectivity needs to be taken into account. Since the application of catalytic materials aims at lower temperatures, which in principle favor incomplete decomposition, the intermediates evolving on the catalytic surface and the corresponding product selectivity gain additional relevance.

## 4 The Role of Catalysis in the Remediation of Gaseous PFAS Emissions

The rapid environmental accumulation of PFAS that are not degradable – neither biologically nor abiotically – is generally considered particularly critical. Consequently, the majority of recent research primarily addresses monitoring, abatement, and mitigation of PFAS dissemination in water and soil,[^53^, ^54^] e.g., as caused by PFAS leaching from products and solid waste. However, due to the high volatility of gaseous PFAS, those are equally important. Notably, while bioremediation of PFAS that already accumulated in the environment is a measure that takes effect only once the environment is already contaminated, dedicated measures for efficient control of gaseous PFAS emissions may prevent undesired PFAS discharge.

The current landscape of catalysis research on gas phase PFAS decomposition appears scattered with several areas remaining unexplored. Different catalytic concepts exist currently or are envisioned for the abatement of gaseous PFAS. Yet, within these concepts, there is a substantial lack of systematic studies that currently hinders the comparison of catalysts and catalytic systems to each other with respect to their performance. The following paragraphs report on the poor data availability and reveal that there are significant gaps within the material and process space.

**Figure 2:**
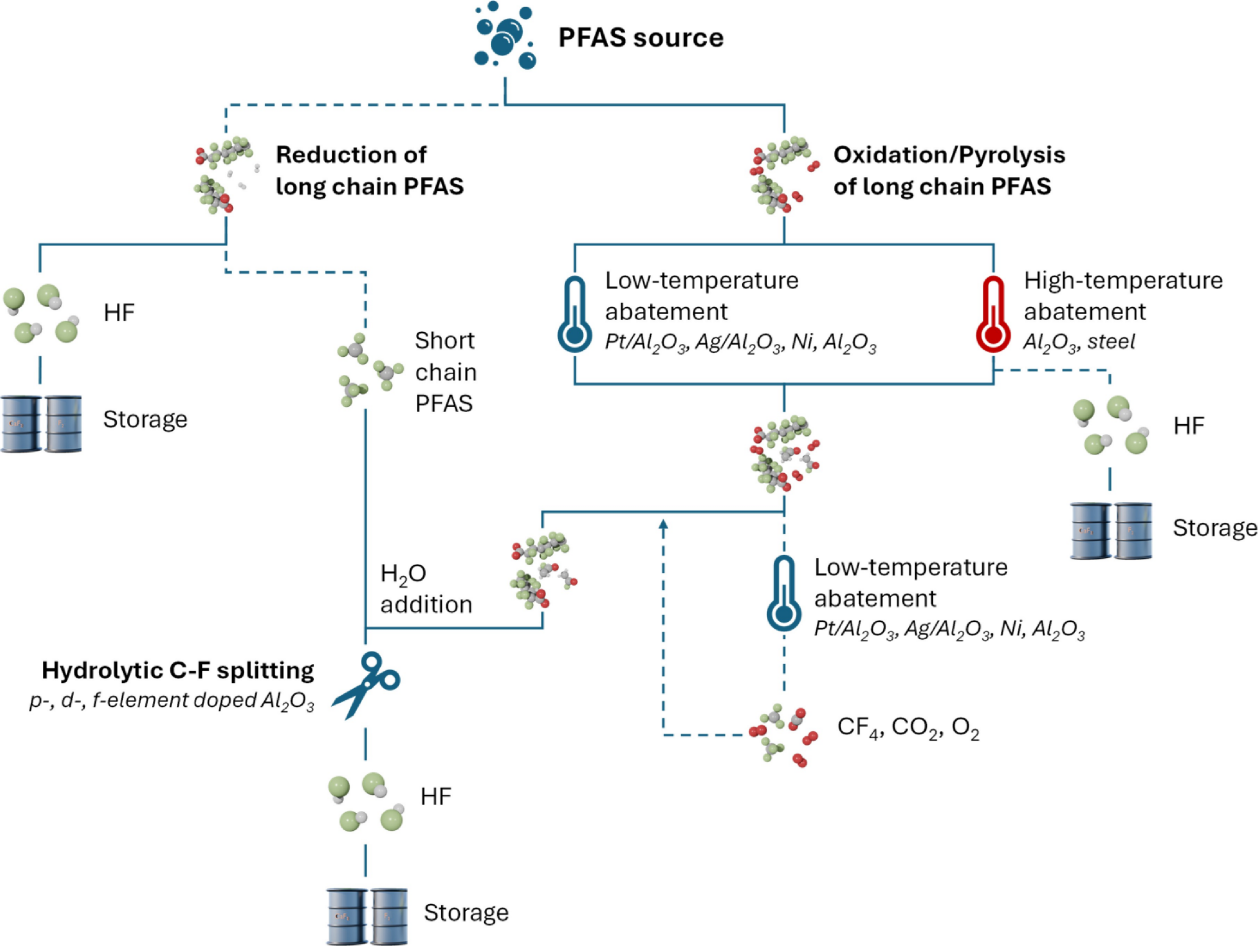
Catalytic routes of PFAS decomposition and mineralization. If applicable, catalytic systems suggested in literature are given at corresponding steps. Dashed lines indicate processes for which there are as yet no or insufficient studies.

The decomposition of PFAS is normally initialized by the non‐fluorinated functional group, which can occur at rather low temperatures, while C−F cleavage and hydrodefluorination is usually considered the rate‐limiting and last step for the mineralization.[^55^, ^56^, ^57^] Therefore, small chain molecules and intermediates from long chain PFAS decomposition are particularly challenging for thermal decomposition. Due to the heterogeneity of PFAS, different approaches are followed including high‐ and low‐temperature treatments under oxidative, inert, and reductive conditions. These steps are brought together in Figure 2 giving a schematic workflow for PFAS abatement in gas phase.

Thermal abatement of PFAS can be conducted in the presence or absence of oxygen, which will be directly determined by the upstream process, e.g., emissions from fine chemistry or incineration. PFAS incineration or pyrolysis is normally conducted at temperatures up to 700 °C (low‐termperature regime) or up to 1000 °C and higher (high‐temperature regime). For oxygen‐free streams, also a reductive abatement in the presence of $H_{2}$ could be possible. In a consecutive step, catalytic C−F splitting of short chain PFAS such as ${\mathrm{CF}}_{4}$ under hydrothermal conditions should be considered as currently no one‐step process yields a 100 % defluorination efficiency. Lastly, quantitative trapping and storage of HF could be achieved as fluorides in an aqueous medium and especially in alkaline earth solutions, e.g. in industrially applied gas scrubbers.[^58^, ^59^]

### 4.1 Oxidation and Pyrolysis of Long Chain PFAS

#### 4.1.1 Low‐Temperature Decomposition and Mineralization

Due to the strong C−F and C−C bonds, gas phase PFAS tend to decomposition rather than mineralization at temperatures below 700 °C.[^50^, ^60^, ^61^] In this context, $C_{1-4}$ ‐PFAS were reported to be the main reaction products, which can be scavenged by the addition of Ca compounds.^[62]^ Thus, different types of catalysts were investigated, from bulk oxides and metals to supported coin and noble metal nanoparticles. Already in the middle of the 20th century, it was found that the combustion of $C_{2}F_{4}$ was enhanced when conducting the reaction in a Ni vessel compared to a steel or borosilicate glass.[^63^, ^64^] Related, also the usage of ${\mathrm{Al}}_{2}O_{3}$ ‐based reactors influenced the reaction rate positively compared to steel[^65^, ^66^] and led to the formation of Al−F‐compounds and ${\mathrm{CO}}_{2}$ under oxygen‐free conditions.[^67^, ^68^] Here, decomposition of perfluorooctanoic acid was reported to start already at temperatures as low as 400 °C; however, full mineralization was not observed even at temperatures of 1000 °C.^[69]^ So far, only few studies applied supported coin or noble metal nanoparticles for PFAS decomposition or mineralization. While a Pd/${\mathrm{Al}}_{2}O_{3}$ ‐based catalyst did not improve the decomposition rate of perfluorohexane at 450 °C,^[70]^ Pt seems to accelerate the initial cracking and C−C splitting, but not mineralization. For the pyrolysis of $C_{2}F_{6}$ , bulk Pt promotes the decomposition at elevated temperatures above 1000 °C.^[71]^ For the decomposition of heptafluorobutyric anhydride, temperatures of 230–350 °C are sufficient when applying a 0.5 % Pt/${\mathrm{Al}}_{2}O_{3}$ catalyst.^[72]^ A similar temperature of 200 °C was also reported for the catalytic combustion of perfluorocarboxylic acids to ${\mathrm{COF}}_{2}$ over a commercial Pt catalyst.^[61]^ In contrast to Cu(111) and Au(111), decomposition of perfluoropentacene multilayers adsorbed on an Ag(111) surface was found to start at temperatures as low as 170 °C. Higher temperatures were even claimed to initiate their partial mineralization,^[73]^ while a 7 % Ag/${\mathrm{Al}}_{2}O_{3}$ catalyst was yielding lower rates for the decomposition of heptafluorobutyric anhydride compared to a Pt‐based one.^[72]^

#### 4.1.2 High‐Temperature Decomposition and Mineralization

At elevated temperatures of 1000 °C and above, mainly the non‐catalytic decomposition of PFAS was in focus of research. Under pyrolysis conditions, most studies agree that long‐chain PFAS start to decompose into ${\mathrm{CO}}_{2}$ at 700 °C,^[60]^ but efficient mineralization takes place only at higher temperatures.^[74]^ Even at temperatures above 1200 °C, slip of ${\mathrm{CF}}_{2}$ , ${\mathrm{CF}}_{4}$ , $C_{2}F_{6}$ , and $C_{2}F_{4}$ is predicted in the product stream besides the desired decomposition products HF, ${\mathrm{CO}}_{2}$ , and CO.^[61]^

Furthermore, Gehrmann et al. investigated PFAS abatement of commercial fluoropolymers in a pilot plant under incineration conditions.^[43]^ In the temperature window up to 1095 °C, only trace amounts of fluorine‐containing compounds were detected due to the ${\mathrm{SiO}}_{2}$ ‐ and ${\mathrm{Al}}_{2}O_{3}$ ‐containing reactor walls scavenging F^−^. At slightly higher reaction temperatures of 1000–1400 °C, a large number of ${\mathrm{CF}}_{2}$ and ${\mathrm{CF}}_{3}$ radicals as result of the gas phase decomposition was theoretically predicted,^[75]^ but, only low concentrations of ${\mathrm{CF}}_{4}$ were experimentally measured. Again, heterogeneous catalytic reactions with the ${\mathrm{Al}}_{2}O_{3}$ ‐containing reactor walls were suggested to increase the reaction rates and could be responsible for the recombination of $C_{1}$ to $C_{2}$ fluoro‐organic molecules. Particularly in oxygen‐ and water‐containing conditions and for temperatures above 900 °C, full mineralization of perfluorooctanoic acid was reported for α ‐${\mathrm{Al}}_{2}O_{3}$ ‐based reactors.^[76]^

### 4.2 Reduction of Long Chain PFAS

So far, the reductive abatement of PFAS and related molecules was considered for liquid phase only. Still, implications can be drawn for a potential application in gas phase from the current available literature. Long et al. published several papers[^77^, ^78^, ^79^, ^80^] tackling the reductive perfluorooctanoic acid abatement using a continuous membrane reactor and platinum group metal nanoparticles as catalyst. Under the given conditions, Pd nanoparticles outperformed Rh, Ru, and Pt regarding the defluorination rate by a factor of approx. 15.^[78]^ H_2_ transfer through the membrane was found to positively influence the observed overall activity and enhanced the hydrodefluorination.^[79]^ Lastly, also bimetallic PdRh nanoparticles with different metal ratios were investigated.^[80]^ While the perfluorooctanoic acid decomposition could not be improved compared to monometallic Pd nanoparticles, increased hydrodefluorination kinetics were observed for bimetallic ones. As the highest observed specific defluorination ratio was approx. 50 %, partly defluorinated or smaller PFAS molecules are likely to be generated.

### 4.3 Hydrolytic C−F Splitting of Short Chain PFAS

The defluorination of short chain perfluorinated carbon molecules is regarded as the most challenging step in the overall decomposition due to the strong C−F bond. In this regard, C−F splitting by hydrolysis over ${\mathrm{Al}}_{2}O_{3}$ ‐based catalysts is considered state‐of‐the‐art and was investigated over the past decades.^[81]^ Different surface modifications including the doping with p‐, d‐, and f‐elements have been considered mainly with the target to increase the long‐time stability and activity at temperatures below 600 °C.

Recently, Zhang et al. postulated three‐coordinated Al (Al^III^) on the γ ‐${\mathrm{Al}}_{2}O_{3}$ (110) surface as the dominant active site.^[82]^ The necessity for hydroxyl groups on the catalyst surface was reported by Ng et al.^[68]^ and also Luo et al.^[83]^ reported on the critical role of hydroxylated alumina sites during catalytic ${\mathrm{CF}}_{4}$ decomposition. Notably, an increase in activity and phase stability was observed when applying alumina phosphates instead of pure alumina.[^84^, ^85^, ^86^] Further addition of light rare earth metals increased the activity yielding ~80–90 % ${\mathrm{CF}}_{4}$ decomposition at 700 °C.

Spinell formation by the addition of Ni or Zn,^[87]^ pre‐treatment by $H_{2}{\mathrm{SO}}_{4}$ ,^[88]^ as well as doping ${\mathrm{Al}}_{2}O_{3}$ with Zr^[89]^ or Ga^[90]^ was reported to increase the amount of Lewis acid sites, which are claimed to be the active sites for C−F splitting.

Besides the activation of the C−F bond, the poisoning of surface sites by fluorine ions is a major challenge. One possible solution is to add scavenger substances, such as Ca(OH)_2_, either directly to the catalyst formulation^[62]^ or as separate beds in multi‐stage reactors.^[91]^ The addition of Ga on the other hand was found to assist the defluorination of poisoned Al active sites increasing the long‐term activity for complete ${\mathrm{CF}}_{4}$ decomposition at 600 °C.^[92]^

### 4.4 Theory‐Guided and Knowledge‐Driven Catalytic Solutions

Given the number of substances classified as PFAS and the typically complex decomposition mechanisms, it can be expected that even comprehensive experimental measurement campaigns will not cover the tremendous variety of PFAS and their fragments potentially evolving during thermal and catalytic degradation. Thus, conceptual approaches and workflows that are established in conventional emission control can inspire holistic theory‐guided workflows in the field of PFAS degradation and remediation. Also, bringing the expertise and methodology of various fields together will be key to success.

The comprehensive knowledge on fluorine chemistry that is available in inorganic and organic chemistry should be exploited first. Amending this know‐how with fundamental ab‐initio data, numerical simulations, and rationally designed experiments that are conducted under well‐defined yet realistic conditions as a next step will enable developing PFAS decomposition mechanisms that rely on both experimental and theoretical knowledge. For instance, a study led by the US EPA investigated the destruction efficiency and the formation of products of incomplete combustion for three PFAS, namely ${\mathrm{CF}}_{4}$ , CHF_3_, and $C_{2}F_{6}$ combining numerical simulations and a pilot‐scale research combustor.^[75]^ This approach can serve as a blueprint for future studies.

In this context, progress has been made recently in understanding PFAS degradation in general and their decomposition and oxidation in the gas phase in particular by means of computational methods. For instance, Reactive Force Field (ReaxFF) molecular dynamics simulations were used to predict the products of incomplete destruction evolving during PTFE pyrolysis, namely $C_{2}F_{4}$ , $C_{2}F_{6}$ , and $C_{3}F_{6}$ .^[93]^ Equally important, several recent theoretical studies on the thermochemistry of PFAS unraveled the complex reaction network of oxidation and decomposition pathways.[^94^, ^95^, ^96^] Herein, first‐principles may be exploited to accurately predict the enthalpies of formation for PFAS, which can then be used during the development of detailed kinetic mechanisms.^[97]^

Beyond understanding PFAS degradation pathways, computational chemistry, i.e. density functional theory (DFT) calculations, can also facilitate finding promising materials that may serve as efficient catalysts for activating the stable C−F bond. For instance, Fe was recently suggested as a metal of particular interest for catalytic degradation of perfluorobutanoic acid, because DFT calculations yielded a good balance of fluorine and perfluorobutanoic acid binding and reaction energies.^[98]^

## 5 Roadmap for Developing Research Targets for Catalytic PFAS Removal

**Figure 3:**
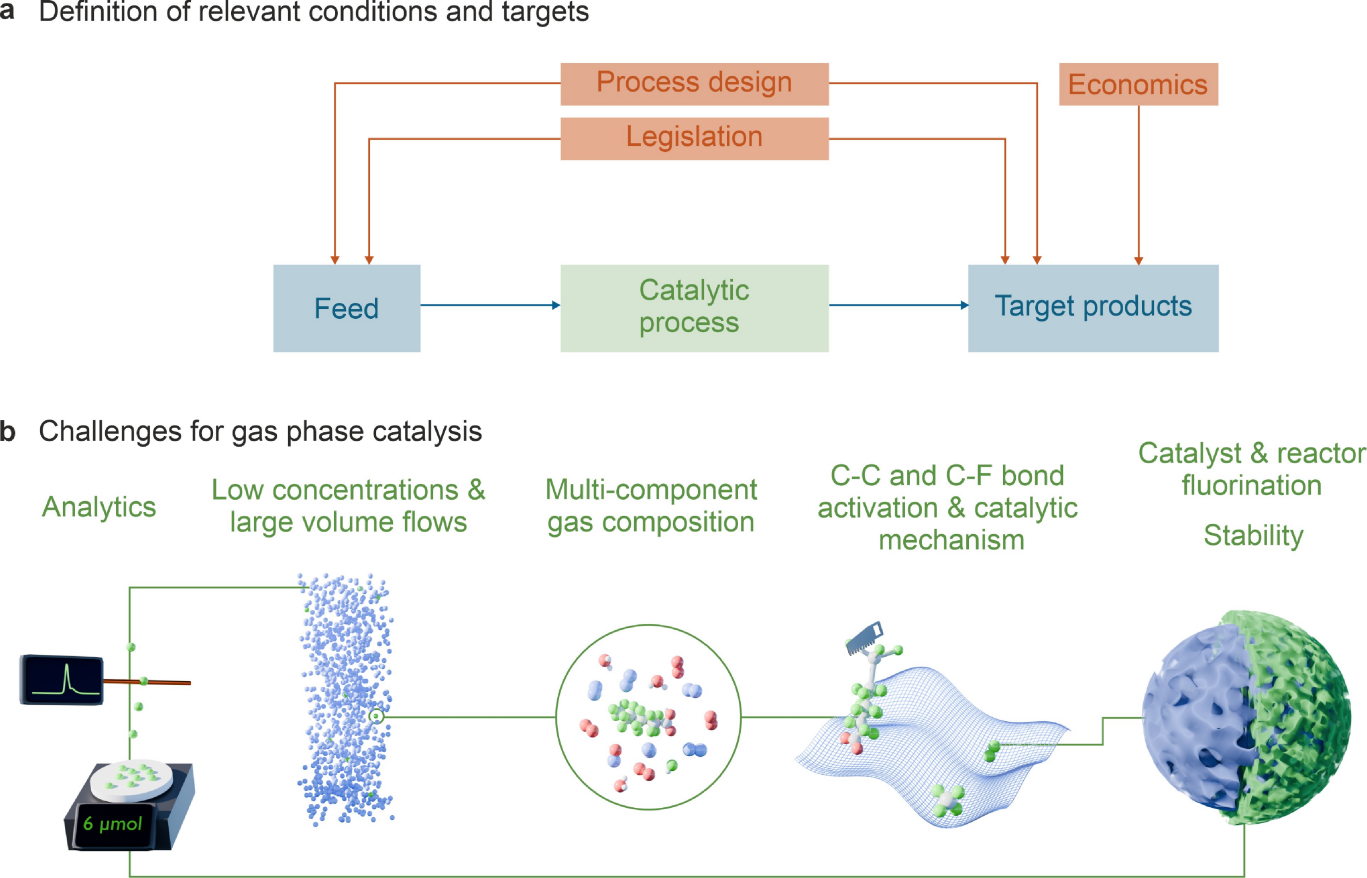
a) Roadmap for research targets in catalytic PFAS removal in the gas phase. b) Critical challenges for catalytic PFAS decomposition.

As demonstrated in previous sections, strategies for the remediation of PFAS can be highly complex. Furthermore, the available data on gas phase catalysis is scattered, concise strategies are missing, and certainly, most PFAS emission sources will require a unique engineering solution. However, to concentrate the scientific efforts and arrive at joint strategies for the field, we believe that process boundaries should be investigated and clarified first (Figure 3a).

### 5.1 F‐Containing Feed Composition

As the catalog of PFAS molecules is huge, research cannot afford to investigate all molecules and their catalytic abatement trajectory. Instead, a range of model compounds and mixtures thereof need to be proposed. In addition, it is important to quantify and indicate which concentrations of these compounds will be the core target for catalytic applications.

Therefore, the pilot plant and lab‐scale experiments for incineration and pyrolysis on realistic PFAS‐containing soil and waste are crucial to determine what might be relevant.^[43]^ Likewise, analysis of other gas phase PFAS sources, like landfill gas under realistic conditions,^[40]^ is key for shaping the feed composition that should be investigated for catalytic applications. Predictive computational investigations can provide additional guidance.

Certainly, categorizing PFAS by terminal functional groups has to be made and initially investigated separately (e.g., S‐containing PFAS, or OH‐terminated functional groups). Further, we propose that within fundamental research small chain fully flourinated alkanes like ${\mathrm{CF}}_{4}$ and $C_{2}F_{6}$ inherit a central role. These are some of the most stable perfluorocarbons,^[99]^ have thus been suggested as particularly feasible model molecules for investigating incineration,^[75]^ and are expected to be the primary non‐mineralized PFAS products exiting pyrolysis and incineration.^[43]^ In addition, these two molecules are relatively simple in terms of what products can be formed and, therefore, will allow the determination of reaction kinetics and enhance understanding of fundamental surface catalysis.

### 5.2 Targeted F‐Containing End Product

In comparison to other emission control reactions, the end products of the PFAS decomposition will contain F and as such should not be released into the environment (in contrast to, for example, ${\mathrm{CO}}_{2}$ or $N_{2}$ ). At this point, it is important to differentiate the different terms used for PFAS decomposition: *Mineralization* of PFAS refers to the complete removal of fluorine atoms, regardless of whether the carbon structure is fully oxidized to ${\mathrm{CO}}_{2}$ . In contrast, *defluorination* involves the release of inorganic fluorine; however, the remaining organic compounds may still exhibit terminal F groups. Lastly, *degradation* refers to the transformation of a target PFAS into a different molecule, which does not necessarily involve defluorination.

In this framework, the decision on target products has an environmental/regulatory and an economic dimension. Production sites handling PFAS synthesis may have the potential to break the emitted molecules to building block monomers, which – if recycled – present a benefit over mineralization. Similarly, if captured before the release into the environment, also small PFAS (i.e., ${\mathrm{CF}}_{4}$ and $C_{2}F_{6}$ ) would present a valuable product. However, most likely, the targeted route for most applications focuses on mineralization. Mineralization will in many cases yield poisonous and reactive HF, which presents an enormous challenge as it should not be released into the environment. Here, further gas treatments will be required to capture HF in water as hydrofluoric acid or to react it with metal ions (like Ca^2+^) to insoluble salts.

### 5.3 Process Design and Integration

Lastly, the catalytic reactor is embedded into a larger process within the PFAS emission source (e.g., a waste incineration facility). This overall process strongly determines the possibilities that exist for the catalytic application, for example, the degree of other gases (e.g., O_2_, H_2_O etc.) that will be present in the process or that potentially could be fed into the catalytic process (e.g., H_2_). Further, the overall process determines whether the PFAS emission control operates under static conditions or is subject to strong fluctuation with respect to concentrations, flow rates, and other species. Consequently, the process integration and consideration for viable overall operation will strongly determine the framework of the catalytic process.

For these reasons, we consider advancement in understanding and the consequent publication of potential boundary conditions as an urgently needed guideline for catalysis research for gas phase PFAS remediation. Also, these boundary conditions will ultimately determine, whether a catalytic emission control technology will make it into a real application or whether other non‐catalytic approaches like pure incineration^[43]^ will ultimately be employed.

## 6 Overarching Challenges

Despite the current lack of defined boundary conditions, the analysis of the existing scientific literature reveals a number of challenges (Figure 3b) that need to be addressed in a catalytic solution and require missing fundamental understanding, engineering and technical solutions.

### 6.1 Reliable Analysis and Closing the F‐Balance

Laboratory research and industrial application require reliable and cost‐effective detection and quantification of F‐species. The methods applied to date are usually not sensitive to all possibly formed F‐containing molecules; therefore, often only degradation efficiencies of target compounds are reported.^[100]^ Furthermore, if attempted to measure all F‐containing species, it remains a challenge to close the F‐balance due to the incorporation of F into the reactor equipment and the significant F content present in the analysis and laboratory equipment itself.

The field would substantially profit from the development of easily accessible analysis tools for full quantification of all F compounds, e.g. by establishing databases for gas phase PFAS, similar as suggested for infrared (IR) spectra of fluorocarbon species.^[101]^ This would facilitate reliable identification and quantification of PFAS, which beyond lab‐scale research is also a prerequisite for the technical realization of any measure considered in this article.

On the fundamental research level, we suggest studying small molecules like ${\mathrm{CF}}_{4}$ as a model molecule because the simplicity of the reaction network will facilitate closing F‐balances and the determination of the exact whereabout of product species.

### 6.2 Low PFAS Concentrations and Mass Transport

In almost any scenario, the target PFAS concentrations will be very low: Depending on the source, the concentration of small chain PFAS may be between 10–10’000 ppb.[^40^, ^43^] Besides the challenge for reliable analytics at these levels, this also represents a challenge for the catalyst itself, due to unfavorable adsorption kinetics as well as the reactor engineering. High volume flows with low concentrations need to be treated, resulting in the demand for low pressure drop solutions and the optimization of mass transport within the catalytic body.

### 6.3 Multi Component Gas Phase Abatement

Especially when dealing with off‐gas from different sites (incineration, pyrolysis, landfills etc.), a range of species rather than a single PFAS molecule will be present, frequently involving not only isolated PFAS, but also species such as NO_x_, hydrocarbons, H_2_O, CO, and CO_2_. As commonly known from conventional exhaust gas catalysis, the interaction of such many different gas species can result in a multitude of side reactions that yield undesired byproducts as well as in an inhibition of the catalytic reaction by blockage of catalytically active (surface) sites. Especially, the strong catalyst poison sulfur may occur during PFAS decomposition.^[51]^ Due to the severity of sulfur poisoning even of noble metal‐based catalysts, such aspects must be taken into account when designing catalyst formulations or catalyst operation procedures. Also humidity in gas streams is a highly relevant factor, for instance as highlighted by Wang et al.^[102]^ who found adsorption energy variations depending on HF and OH concentrations on γ ‐${\mathrm{Al}}_{2}O_{3}$ (110) surfaces.

### 6.4 Catalytic Activation of C−F and C−C Bonds

As mentioned above, typical PFAS molecules consist of a perflourinated carbon chain with a functionalized head group. While the activation of the head group is normally easily achieved, the C−C and in particular C−F bonds are the critical step in the abatement of PFAS.[^83^, ^99^]

In a recent, theoretical study by Blotevogel et al.,^[103]^ the cleaveage of the C−C bond was found to be the kinetic bottleneck for PFAS thermal remediation of various PFAS at temperatures above 700 °C. In homogeneous catalysis, highly oxidizing species such as hydroxyl, peroxide or sulfate radicals have to be added to cleave these. In gas phase, the high thermal stability leads to the need to apply temperatures up to 1100 °C, at which O_2_ or H_2_O are able to decompose PFAS effectively. First studies could already show that breaking up the carbon‐carbon backbone of the PFAS molecules requires up to ~950 °C at 2 s gas residence time, making this step the kinetic bottleneck on the way to complete thermal PFAS mineralization.^[103]^

Activation of these bonds in gas phase and bringing the temperature down is therefore of utmost importance if such processes should be ever applied in large scale. However, fundamental mechanistic understanding of the functioning and mechanisms of the catalytic transformation of PFAS is essentially missing. Only a small variety of different materials have been tested and knowledge on, e.g., scaling relations of C−F bond activation to catalyst surface structures remain unexplored. The field of materials which potentially could be employed remains huge and systematic understanding of elementary reaction steps like adsorption, bond breaking, and desorption remain open do debate. Understanding this network is particularly important for controlling the catalyst selectivity, as PFAS decomposition bears the potential of producing products that are harmful and toxic.

### 6.5 Catalyst Stability and Reactor Fluorination

One of the major reasons why catalytic tests are unable to close the F‐balance originates from fluorination of the catalyst and reactor material. The breaking of the C−F bond on a catalytic surface results in the formation of a new strong F‐bond with the catalytic surface. Often this bond is significantly stronger than the C−F bond. For example, the Al−F bond strength^[104]^ is ~660 kJ mol^−1^ leading to the formation of fluorinated catalyst surfaces.^[92]^

This fluorination often leads to rapid deactivation of catalytic materials, which poses an additional challenge compared to conventional emission control catalysis, and requires periodic catalyst regeneration. In this regard, detailed knowledge on the surface reaction mechanism is key.

Two ways of stabilizing catalyst materials are currently being discussed, either by controlling the reaction conditions or by depositing fluorine‐resistant/defluorination‐promoting elements. For instance, based on ab initio molecular dynamics simulations and *in situ* experiments, Luo et al.^[83]^ report that ${\mathrm{CF}}_{4}$ decomposition over ${\mathrm{Al}}_{2}O_{3}$ involves lattice oxygen and that a sufficient supply of hydroxyl groups enables to refill oxygen vacancies and thus an efficient C−F bond cleavage. As an alternative, Zhang et al.^[92]^ doped an alumina catalyst with gallium, because the Ga−F bond is weaker than the fairly stable Al−F bond, which facilitates hydrolysis to Ga‐OH that then assists the defluorination of F‐poisoned Al‐sites. Consequently, investigating the material‐specific mechanism of fluorination^[105]^ and the consequences for the catalytic process are a key challenge and require fundamental research.

## 7 Conclusion

Due to the omnipresence of PFAS, their treatment will become and remain a major issue throughout their life‐cycle and in the context of material recycling. In the end, not solely scientific and technical questions will decide on the fate of PFAS emission abatement, but also economic and legislative considerations will come into play.

In this respect, the remediation of PFAS in dispersed polluted sources (water, soil, and ambient air) with comparably low PFAS concentrations will likely cause much higher costs than the abatement of PFAS emissions from specific sources, such as production sites, incineration plants, or landfills, where concentrations are higher and the origin is typically limited to a single effluent gas stream.

As outlined in this minireview, prospects for resolving these issues by means of catalytic solutions exist. Yet, system design and fundamental understanding on the topic are in their infancy in comparison to established emission control. In order to provide the society with technically and economically attractive solutions for full‐scale applications, different disciplines, such as inorganic chemistry, catalysis, chemical engineering, and material sciences, need to join forces.

The approaches reviewed herein allow an encouraging conclusion: With a concerted effort across different scientific‐technical disciplines, air pollution with the forever‐chemicals PFAS will not be an eternal problem, but can be tackled with dedicated catalysts.

## Conflict of Interests

The authors declare no competing interest.

8

## Biographical Information

*Patrick Lott leads the “Catalytic Reactors” group at the Institute for Chemical Technology and Polymer Chemistry and is Chief Technology Officer at the Center for Emission Control Karlsruhe at the Karlsruhe Institute of Technology (KIT), Germany. With his research he aims at developing, understanding, and improving chemical reactors and processes for reducing local and global pollutants by means of in situ techniques that provide insights into chemical reactors with spatial and temporal resolution and allow for boosting the performance of heterogeneous catalysts as well as process optimization*.

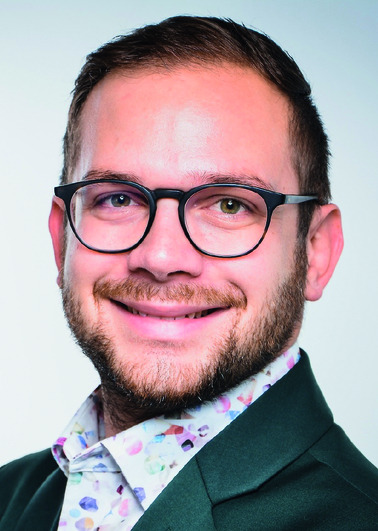

## Biographical Information

*Florian Maurer is a chemist specializing in catalyst research and spectroscopy, with a focus on noble‐metal‐based environmental catalysts. His work leverages advanced in situ and operando spectroscopy techniques, particularly using hard X‐rays, to pinpoint and monitor active sites in heterogeneous catalysts under real‐world conditions. As a postdoctoral fellow at the Karlsruhe Institute of Technology (KIT), he also coordinates the Collaborative Research Center CRC1441 “TrackAct” in which innovative concepts for catalysts and their characterization are developed*.

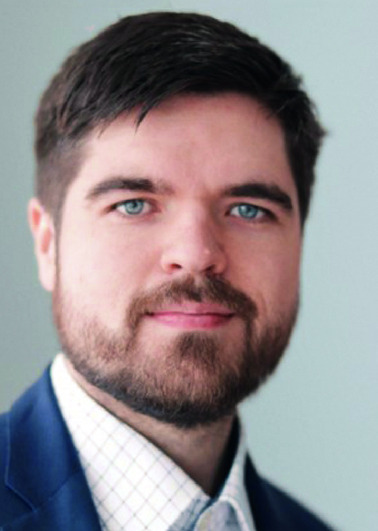

## Biographical Information

*Arik Beck is a chemical engineer by training and a surface scientist at heart. He leads a Young Investigator Group at the Karlsruhe Institute of Technology (KIT), where his research focuses on unraveling the transformation processes of heterogeneous catalysts under reactive environments. By employing advanced operando characterization tools, his group aims to deepen the understanding of rapid material transformations and their kinetics. A particular focus of his work is leveraging these insights to pioneer novel catalytic applications*.

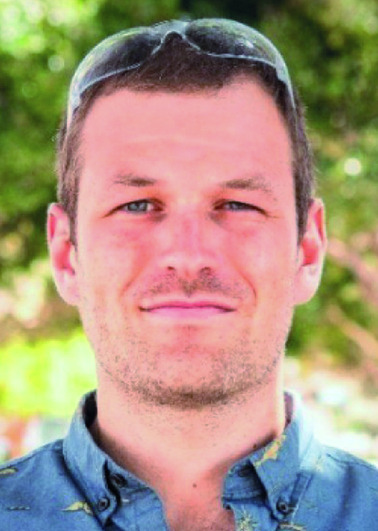

## Data Availability

Data sharing is not applicable to this article as no new data were created or analyzed in this study.
